# Supplementary material for: Neutralizing Antibody Responses After mRNA COVID-19 Booster Vaccination are Unaffected by Parasitemia in a Malaria-Endemic Setting
Source: medRxiv. 2025 Apr 16:2025.04.12.25325718. Preprint. [Version 1] doi: 10.1101/2025.04.12.25325718 (PMC12047954; doi:10.1101/2025.04.12.25325718)
Supplement: Supplement 1 [file media-1.pdf]

Supplementary Figure S1. Activities during parent study ("Pre-enrolment") in relation to the Kombewa malaria substudy ("Enrolment")

	Pre-enrolment						Enrolment	
	M0	M1	M2	M3	M4	M5	M0	M1
Group 1 SARSC2-* (n=121)							†	
Group 2 SARSC2+* (n=205)							†	
Pf RT-PCR	‡	**						
anti-Spike neut antibody								

NOTE

* 4/330 participants were excluded due to missing malaria tests at Enrolment Month 0

† Randomized 1:1 at Pre-enrolment Month 0 to receive either mRNA-1273 or mRNA-1273.222

‡ Since the parent study (Pre-enrolment period) began enrolment before the malaria sub-study was IRB approved, not all participants had a Pre-enrolment M0 *P falciparum* (Pf-PCR)

** Since Group 1 participants had a baseline negative point-of-care anti-SARS-Cov-2 (SARSC2) antibody test, they returned at Pre-enrolment M1 to receive a second vaccine and were re-tested with Pf-PCR

Supplementary Figure S2. CONSORT diagram showing parent cohort and Kombewa substudy cohort

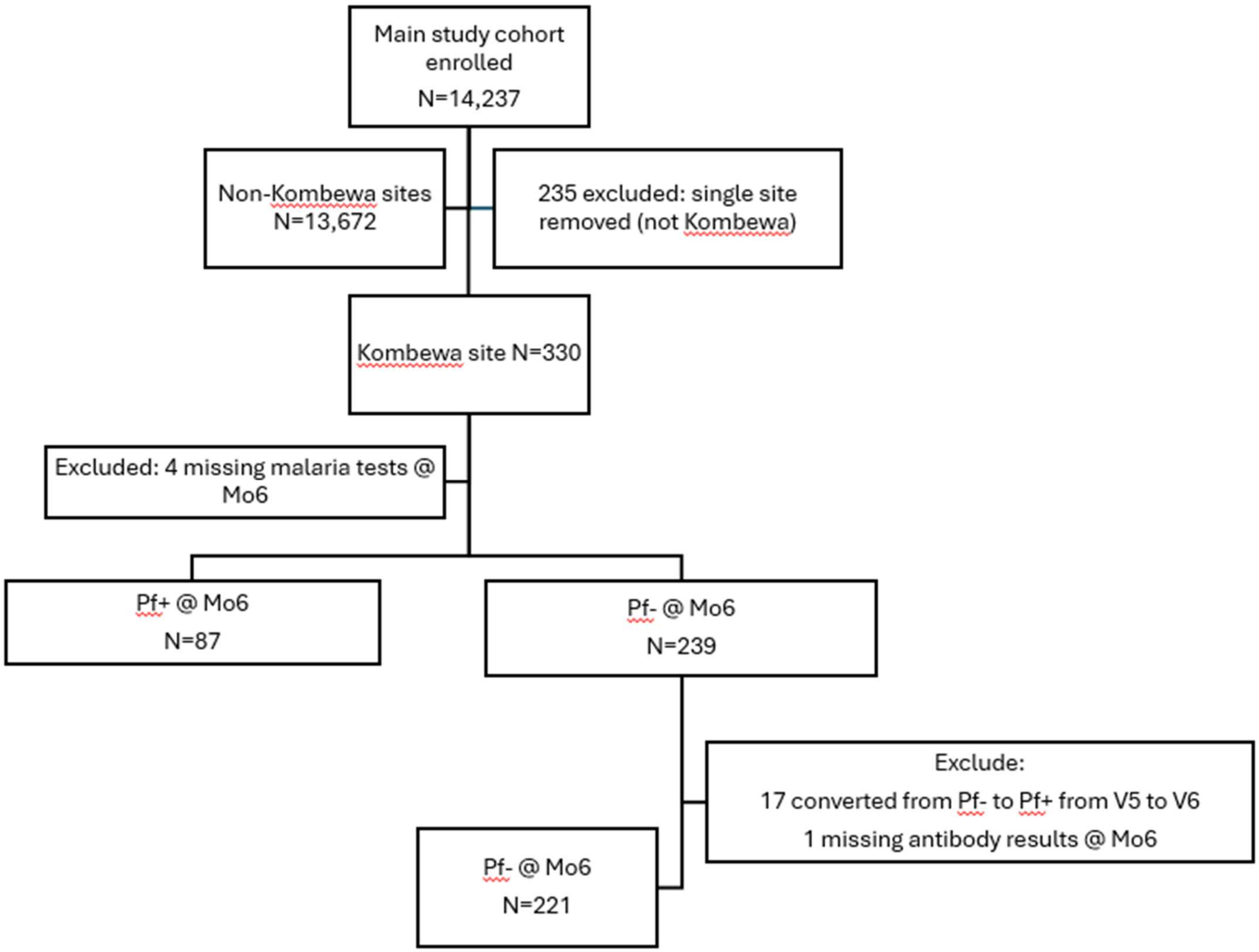

Supplementary Table S1. Baseline characteristics at enrolment (M0) in asymptomatic Pf-PCR-positive (Malaria+) and -negative (Malaria-) participants using the primary objective cohort

Characteristic	Malaria+ at V5, N = 87	Malaria- at V5 and V6, N = 221	Total, N = 308	P-value*
Sex at birth - N (%)				0.0680
Female	55 (63.2%)	163 (73.8%)	218 (70.8%)	
Male	32 (36.8%)	58 (26.2%)	90 (29.2%)	
Age category - N (%)				0.4840
<=40 years	52 (59.8%)	142 (64.3%)	194 (63.0%)	
>40 years	35 (40.2%)	79 (35.7%)	114 (37.0%)	
BMI at baseline (kg/m2) - N (%)				0.0421
<=25	77 (88.5%)	173 (78.3%)	250 (81.2%)	
>25	10 (11.5%)	48 (21.7%)	58 (18.8%)	
CD4 at V5 (cells/mm3) - N (%)				1.0000
<350	6 (6.9%)	18 (8.1%)	24 (7.8%)	
>=350	81 (93.1%)	203 (91.9%)	284 (92.2%)	
HIV viral load at V5 (copies/mL) - N (%)				0.0491
<40	61 (70.1%)	178 (80.5%)	239 (77.6%)	
>=40	26 (29.9%)	43 (19.5%)	69 (22.4%)	
Month 6 vaccination - N (%)				1.0000
mRNA 1273	43 (49.4%)	111 (50.2%)	154 (50.0%)	
mRNA 1273.222	44 (50.6%)	110 (49.8%)	154 (50.0%)	
Month 6 immunity status - N (%)				0.1016
Hybrid	68 (78.2%)	190 (86.0%)	258 (83.8%)	
Vaccine	19 (21.8%)	31 (14.0%)	50 (16.2%)	
Diabetes at baseline - N (%)	0 (0.0%)	0 (0.0%)	0 (0.0%)	1.0000
Chronic kidney disease at baseline - N (%)	0 (0.0%)	0 (0.0%)	0 (0.0%)	1.0000
Cancer at baseline - N (%)	0 (0.0%)	0 (0.0%)	0 (0.0%)	1.0000
Non-HIV immunocompromised state (weakened immune system) or solid organ transplant at baseline - N (%)	0 (0.0%)	1 (0.5%)	1 (0.3%)	1.0000
Autoimmune disease at baseline - N (%)	0 (0.0%)	0 (0.0%)	0 (0.0%)	1.0000
Immunodeficiency at baseline - N (%)	0 (0.0%)	0 (0.0%)	0 (0.0%)	1.0000
Pregnancy at baseline** - N (%)				0.3797
Yes	0 (0.0%)	3 (1.8%)	3 (1.4%)	
No	55 (100.0%)	160 (98.2%)	215 (98.6%)	

Note:

*P-values for comparison between malaria+ and malaria- participants are obtained from Barnard's test.

**Pregnancy at baseline is calculated among females assigned at birth only.

Primary objective cohort: malaria+ if PCR+ at V5; malaria- if PCR- at V5 and V6; ppts not assigned malaria+ or malaria- are excluded.

Participants meet all the following criteria:

1. Received the booster shot at V5 (Month 6).
2. The neutralizing antibody titer was tested at both V5 and V6 and the V6 draw was +/- 14 days of V6, where V6 is 28 days after V5. In other words, if the V6 titer was drawn <15 days or >42 days after the booster the participant will be excluded.

Supplementary Table S2. ID50 and ID80 geometric mean titers of neutralizing anti-D614G Spike antibody to booster vaccines at M0 (booster receipt) and M1 in asymptomatic Pf-PCR-positive (Malaria+) and -negative (Malaria-) participants using the primary objective cohort

Isolate	Dilution	Visit	Malaria Positivity	N	Geometric Mean Titer (95% CI)	Median (Q1, Q3)	(Min, Max)	P-value*
SARS-Cov-2 D614G	50	5	Malaria- at V5 and V6	221	2765.3 (2331, 3280.5)	2821.3 (1283.1, 6501.8)	(37.7, 159969.3)	0.0844
			Malaria+ at V5	87	2079.7 (1576.8, 2743)	2462.9 (1088.6, 4481.9)	(70.8, 64569.1)	
		6	Malaria- at V5 and V6	221	26931.8 (21778, 33305.1)	28586 (10423.3, 67174.1)	(5, 781250)	0.2704
			Malaria+ at V5	87	22019.1 (16443.6, 29485)	18256.4 (9346.4, 43141.5)	(1231.4, 781250)	
	80	5	Malaria- at V5 and V6	221	763.4 (637.5, 914.2)	826.5 (360.7, 1953.1)	(5, 69596.2)	0.0549
			Malaria+ at V5	87	545.6 (406.9, 731.7)	563.8 (278.4, 1375.1)	(19, 22253.6)	
		6	Malaria- at V5 and V6	221	7730.7 (6270.9, 9530.3)	8146.5 (3120.6, 21474.4)	(5, 781250)	0.1029
			Malaria+ at V5	87	5823.9 (4440.7, 7637.9)	5117.3 (2965, 9320.7)	(365.1, 150863.3)	

Note:
*P-values for comparison of geometric means between malaria+ and malaria- participants are obtained from T-tests performed on log-transformed titer values.
95% confidence intervals are calculated using Student's t-distribution.

Supplementary Figure S3. Violin boxplots of ID80 neutralizing anti-D614G Spike antibody to booster vaccines in asymptomatic Pf-PCR-positive and -negative participants at M0 and M1 (red=monovalent mRNA-1273, blue=bivalent mRNA-1273.222.

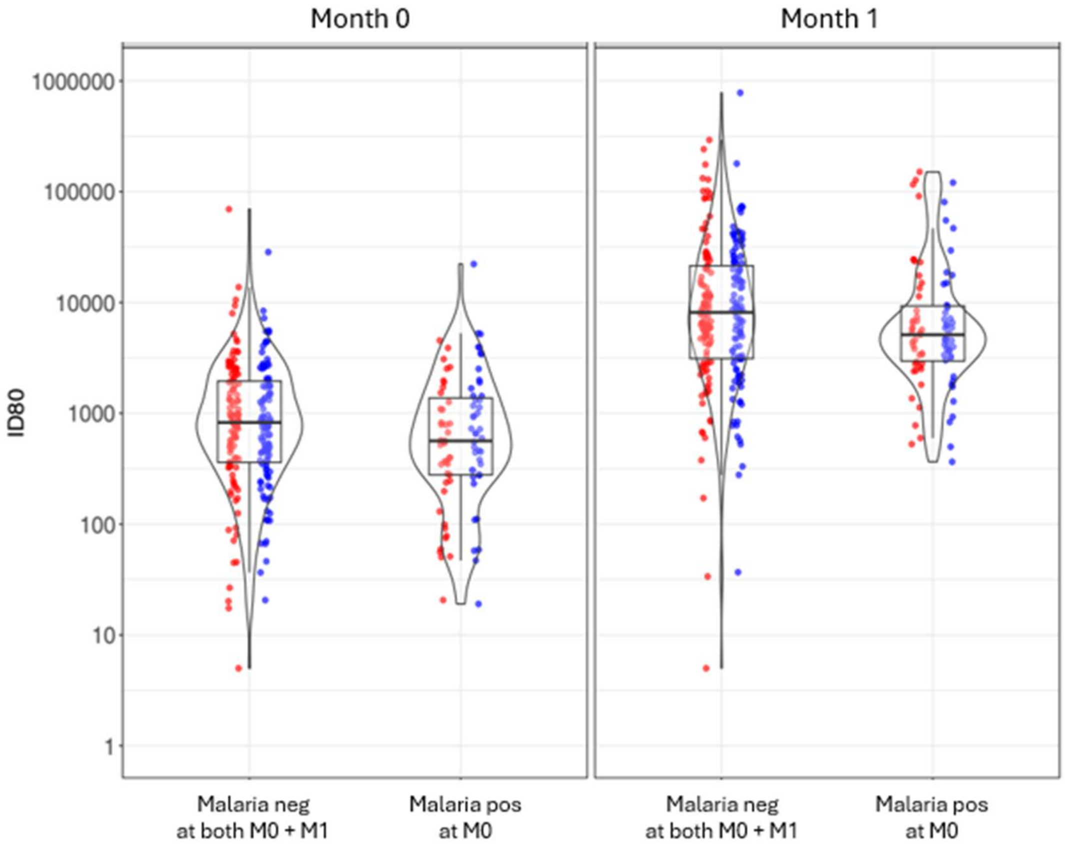

Supplementary Table S3. ID50 and ID80 geometric mean fold rise (M1 over M0) neutralizing anti-D614G Spike antibody to booster vaccines in asymptomatic Pf-PCR-positive (Malaria+) and -negative (Malaria-) participants using the primary objective cohort

Isolate	Dilution	Malaria Positivity	N	Geometric Mean Fold Rise (95% CI)	Median (Q1, Q3)	(Min, Max)	P-value*
SARS-Cov-2 D614G	50	Malaria- at V5 and V6	221	9.7 (7.9, 12)	8.6 (3.7, 25.7)	(0.1, 652.4)	0.6756
		Malaria+ at V5	87	10.6 (7.6, 14.8)	8.4 (4, 22.8)	(0.6, 3263.8)	
	80	Malaria- at V5 and V6	221	10.1 (8.3, 12.4)	9.5 (3.9, 24.9)	(0.1, 427.4)	0.7851
		Malaria+ at V5	87	10.7 (7.7, 14.8)	8.6 (4, 20.2)	(0.6, 1949.4)	

Note:
*P-values for comparison of geometric mean fold rises between malaria+ and malaria- participants are obtained from T-tests performed on log-transformed fold rise values.
95% confidence intervals are calculated using Student's t-distribution.

Supplementary Table S4. Base and univariate models of ID50 and ID80 geometric mean ratio estimate (M1 over M0) comparing Pf-PCR-positive (Malaria+) to -negative participants using the primary objective cohort

Outcome	Model	Covariate	Geometric Mean Ratio Estimate (95% CI)	P-value
ID50 fold rise	Base	malaria_status_pobj1Malaria+ at V5	1.0871 (0.7352, 1.6074)	0.6746
	Univariate 1	malaria_status_pobj1Malaria+ at V5	1.0990 (0.7413, 1.6293)	0.6374
		SEXMale	0.9018 (0.6107, 1.3316)	0.6021
	Univariate 2	malaria_status_pobj1Malaria+ at V5	1.0770 (0.7282, 1.5927)	0.7095
		AGEGR1_f>40 years	1.2327 (0.8559, 1.7754)	0.2600
	Univariate 3	malaria_status_pobj1Malaria+ at V5	1.0760 (0.7253, 1.5962)	0.7151
		BMIBL_f>25	0.9043 (0.5742, 1.4241)	0.6632
	Univariate 4	malaria_status_pobj1Malaria+ at V5	1.0817 (0.7317, 1.5991)	0.6929
		CD4M6_f>=350	1.4901 (0.7728, 2.8732)	0.2328
	Univariate 5	malaria_status_pobj1Malaria+ at V5	1.1221 (0.7574, 1.6622)	0.5646
		HIVVLM6_f>=40	0.7383 (0.4830, 1.1285)	0.1604
	Univariate 6	malaria_status_pobj1Malaria+ at V5	1.0878 (0.7353, 1.6092)	0.6728
		TRT03P_fmRNA 1273.222	0.9266 (0.6513, 1.3184)	0.6709
	Univariate 7	malaria_status_pobj1Malaria+ at V5	1.1047 (0.7457, 1.6367)	0.6184
		SCOV2M6_fVaccine	0.8138 (0.5036, 1.3150)	0.3989
ID80 fold rise	Base	malaria_status_pobj1Malaria+ at V5	1.0541 (0.7240, 1.5346)	0.7829
	Univariate 1	malaria_status_pobj1Malaria+ at V5	1.0619 (0.7275, 1.5500)	0.7550
		SEXMale	0.9322 (0.6411, 1.3556)	0.7125
	Univariate 2	malaria_status_pobj1Malaria+ at V5	1.0407 (0.7153, 1.5143)	0.8342
		AGEGR1_f>40 years	1.3281 (0.9362, 1.8841)	0.1113
	Univariate 3	malaria_status_pobj1Malaria+ at V5	1.0516 (0.7199, 1.5360)	0.7941
		BMIBL_f>25	0.9773 (0.6317, 1.5118)	0.9174
	Univariate 4	malaria_status_pobj1Malaria+ at V5	1.0490 (0.7207, 1.5270)	0.8021
		CD4M6_f>=350	1.4670 (0.7809, 2.7561)	0.2327
	Univariate 5	malaria_status_pobj1Malaria+ at V5	1.0847 (0.7436, 1.5823)	0.6720
		HIVVLM6_f>=40	0.7595 (0.5052, 1.1418)	0.1853
	Univariate 6	malaria_status_pobj1Malaria+ at V5	1.0549 (0.7243, 1.5365)	0.7799
		TRT03P_fmRNA 1273.222	0.9051 (0.6451, 1.2699)	0.5627
	Univariate 7	malaria_status_pobj1Malaria+ at V5	1.0829 (0.7430, 1.5781)	0.6777
		SCOV2M6_fVaccine	0.7080 (0.4470, 1.1213)	0.1405

Note:
Covariates besides malaria status included in univariate models have prevalence >=5% in all categories for the cohort.

Supplementary Table S5. Base and univariate models of geometric mean ratio estimate (M1 over M0 comparing Pf-PCR-positive to -negative participants) using the secondary objective ‘a’ cohort (i.e., considering participants as Pf-PCR-positive if they tested positive at either M0 or M1)

Outcome	Model	Covariate	Geometric Mean Ratio Estimate (95% CI)	P-value
ID50 fold rise	Base	malaria_status_sobj1Malaria+ at V5 or V6	0.8534 (0.5901, 1.2341)	0.3984
	Univariate 1	malaria_status_sobj1Malaria+ at V5 or V6	0.8582 (0.5922, 1.2439)	0.4183
		SEXMale	0.9410 (0.6432, 1.3769)	0.7536
	Univariate 2	malaria_status_sobj1Malaria+ at V5 or V6	0.8470 (0.5857, 1.2250)	0.3766
		AGEGR1_f>40 years	1.2262 (0.8584, 1.7517)	0.2614
	Univariate 3	malaria_status_sobj1Malaria+ at V5 or V6	0.8409 (0.5797, 1.2199)	0.3603
		BMIBL_f>25	0.8657 (0.5535, 1.3539)	0.5262
	Univariate 4	malaria_status_sobj1Malaria+ at V5 or V6	0.8493 (0.5873, 1.2283)	0.3845
		CD4M6_f>=350	1.4007 (0.7342, 2.6721)	0.3055
	Univariate 5	malaria_status_sobj1Malaria+ at V5 or V6	0.8749 (0.6036, 1.2681)	0.4792
		HIVVLM6_f>=40	0.7863 (0.5203, 1.1882)	0.2528
	Univariate 6	malaria_status_sobj1Malaria+ at V5 or V6	0.8551 (0.5910, 1.2371)	0.4050
		TRT03P_fmRNA 1273.222	0.9114 (0.6457, 1.2864)	0.5968
	Univariate 7	malaria_status_sobj1Malaria+ at V5 or V6	0.8597 (0.5936, 1.2452)	0.4228
		SCOV2M6_fVaccine	0.8867 (0.5535, 1.4207)	0.6162
ID80 fold rise	Base	malaria_status_sobj1Malaria+ at V5 or V6	0.8414 (0.5897, 1.2006)	0.3401
	Univariate 1	malaria_status_sobj1Malaria+ at V5 or V6	0.8439 (0.5902, 1.2068)	0.3514
		SEXMale	0.9688 (0.6713, 1.3982)	0.8652
	Univariate 2	malaria_status_sobj1Malaria+ at V5 or V6	0.8331 (0.5842, 1.1881)	0.3123
		AGEGR1_f>40 years	1.3101 (0.9296, 1.8463)	0.1224
	Univariate 3	malaria_status_sobj1Malaria+ at V5 or V6	0.8338 (0.5825, 1.1935)	0.3195
		BMIBL_f>25	0.9144 (0.5941, 1.4074)	0.6833
	Univariate 4	malaria_status_sobj1Malaria+ at V5 or V6	0.8378 (0.5871, 1.1956)	0.3283
		CD4M6_f>=350	1.3595 (0.7294, 2.5336)	0.3326
	Univariate 5	malaria_status_sobj1Malaria+ at V5 or V6	0.8622 (0.6029, 1.2329)	0.4153
		HIVVLM6_f>=40	0.7904 (0.5310, 1.1767)	0.2458
	Univariate 6	malaria_status_sobj1Malaria+ at V5 or V6	0.8431 (0.5906, 1.2036)	0.3463
		TRT03P_fmRNA 1273.222	0.9124 (0.6546, 1.2718)	0.5876
	Univariate 7	malaria_status_sobj1Malaria+ at V5 or V6	0.8549 (0.5986, 1.2210)	0.3876
		SCOV2M6_fVaccine	0.7730 (0.4912, 1.2166)	0.2649

Note:
Covariates besides malaria status included in univariate models have prevalence >=5% in all categories for the cohort.

Supplementary Table S6. Base and univariate models of geometric mean ratio estimate (M1 over M0 comparing Pf-PCR-positive to -negative participants) using the secondary objective 'b' cohort (i.e., excluding Pf-PCR-negative participants if they had tested positive 4-5 months prior to substudy enrolment)

Outcome	Model	Covariate	Geometric Mean Ratio Estimate (95% CI)	P-value
ID50 fold rise	Base	malaria_status_sobj2Malaria+ at V5	1.0675 (0.6989, 1.6306)	0.7617
	Univariate 1	malaria_status_sobj2Malaria+ at V5	1.0872 (0.7092, 1.6667)	0.7002
		SEXMale	0.8640 (0.5714, 1.3064)	0.4869
	Univariate 2	malaria_status_sobj2Malaria+ at V5	1.0639 (0.6964, 1.6253)	0.7738
		AGEGR1_f>40 years	1.2065 (0.8138, 1.7888)	0.3486
	Univariate 3	malaria_status_sobj2Malaria+ at V5	1.0627 (0.6940, 1.6272)	0.7790
		BMIBL_f>25	0.9410 (0.5678, 1.5595)	0.8127
	Univariate 4	malaria_status_sobj2Malaria+ at V5	1.0554 (0.6911, 1.6117)	0.8021
		CD4M6_f>=350	1.5796 (0.7990, 3.1228)	0.1877
	Univariate 5	malaria_status_sobj2Malaria+ at V5	1.1143 (0.7284, 1.7046)	0.6166
		HIVVLM6_f>=40	0.6748 (0.4254, 1.0702)	0.0942
	Univariate 6	malaria_status_sobj2Malaria+ at V5	1.0667 (0.6977, 1.6308)	0.7648
		TRT03P_fmRNA 1273.222	1.0427 (0.7104, 1.5305)	0.8302
	Univariate 7	malaria_status_sobj2Malaria+ at V5	1.1127 (0.7284, 1.6998)	0.6201
		SCOV2M6_fVaccine	0.6305 (0.3892, 1.0214)	0.0608
ID80 fold rise	Base	malaria_status_sobj2Malaria+ at V5	1.0099 (0.6700, 1.5223)	0.9622
	Univariate 1	malaria_status_sobj2Malaria+ at V5	1.0232 (0.6764, 1.5480)	0.9132
		SEXMale	0.9009 (0.6035, 1.3449)	0.6085
	Univariate 2	malaria_status_sobj2Malaria+ at V5	1.0055 (0.6673, 1.5149)	0.9792
		AGEGR1_f>40 years	1.2778 (0.8730, 1.8702)	0.2062
	Univariate 3	malaria_status_sobj2Malaria+ at V5	1.0085 (0.6674, 1.5238)	0.9679
		BMIBL_f>25	0.9810 (0.6013, 1.6003)	0.9385
	Univariate 4	malaria_status_sobj2Malaria+ at V5	0.9987 (0.6628, 1.5049)	0.9951
		CD4M6_f>=350	1.5651 (0.8088, 3.0285)	0.1826
	Univariate 5	malaria_status_sobj2Malaria+ at V5	1.0474 (0.6935, 1.5819)	0.8251
		HIVVLM6_f>=40	0.7161 (0.4578, 1.1201)	0.1427
	Univariate 6	malaria_status_sobj2Malaria+ at V5	1.0098 (0.6693, 1.5234)	0.9628
		TRT03P_fmRNA 1273.222	1.0079 (0.6949, 1.4617)	0.9669
	Univariate 7	malaria_status_sobj2Malaria+ at V5	1.0633 (0.7067, 1.6000)	0.7675
		SCOV2M6_fVaccine	0.5639 (0.3541, 0.8978)	0.0160*

Note:
Covariates besides malaria status included in univariate models have prevalence >=5% in all categories for the cohort.

Supplementary Table S7. Base and univariate models of geometric mean ratio estimate (M1 over M0 comparing Pf-PCR-positive to -negative participants) using secondary objective ‘c’ cohort (i.e., excluding Pf-PCR-negative participants if they had tested positive up to 6 months before enrolment)

Outcome	Model	Covariate	Geometric Mean Ratio Estimate (95% CI)	P-value
ID50 fold rise	Base	malaria_status_sobj3Malaria+ at V5	1.0501 (0.6724, 1.6399)	0.8291
	Univariate 1	malaria_status_sobj3Malaria+ at V5	1.0656 (0.6786, 1.6732)	0.7817
		SEXMale	0.9030 (0.5775, 1.4118)	0.6530
	Univariate 2	malaria_status_sobj3Malaria+ at V5	1.0520 (0.6736, 1.6429)	0.8229
		AGEGR1_f>40 years	1.2348 (0.8160, 1.8684)	0.3166
	Univariate 3	malaria_status_sobj3Malaria+ at V5	1.0264 (0.6535, 1.6120)	0.9095
		BMIBL_f>25	0.8386 (0.4991, 1.4090)	0.5044
	Univariate 4	malaria_status_sobj3Malaria+ at V5	1.0460 (0.6697, 1.6338)	0.8425
		CD4M6_f>=350	1.4122 (0.7004, 2.8473)	0.3329
	Univariate 5	malaria_status_sobj3Malaria+ at V5	1.1034 (0.7034, 1.7308)	0.6671
		HIVVLM6_f>=40	0.6984 (0.4202, 1.1609)	0.1652
	Univariate 6	malaria_status_sobj3Malaria+ at V5	1.0500 (0.6716, 1.6416)	0.8297
		TRT03P_fmRNA 1273.222	1.0195 (0.6799, 1.5287)	0.9251
	Univariate 7	malaria_status_sobj3Malaria+ at V5	1.0511 (0.6689, 1.6516)	0.8281
		SCOV2M6_fVaccine	0.9922 (0.5704, 1.7261)	0.9778
ID80 fold rise	Base	malaria_status_sobj3Malaria+ at V5	1.0066 (0.6478, 1.5639)	0.9767
	Univariate 1	malaria_status_sobj3Malaria+ at V5	1.0200 (0.6530, 1.5934)	0.9303
		SEXMale	0.9115 (0.5860, 1.4179)	0.6797
	Univariate 2	malaria_status_sobj3Malaria+ at V5	1.0086 (0.6494, 1.5666)	0.9693
		AGEGR1_f>40 years	1.2706 (0.8440, 1.9128)	0.2498
	Univariate 3	malaria_status_sobj3Malaria+ at V5	0.9915 (0.6344, 1.5496)	0.9700
		BMIBL_f>25	0.8903 (0.5329, 1.4875)	0.6558
	Univariate 4	malaria_status_sobj3Malaria+ at V5	1.0025 (0.6452, 1.5576)	0.9912
		CD4M6_f>=350	1.4381 (0.7192, 2.8757)	0.3025
	Univariate 5	malaria_status_sobj3Malaria+ at V5	1.0428 (0.6675, 1.6291)	0.8531
		HIVVLM6_f>=40	0.7735 (0.4675, 1.2797)	0.3157
	Univariate 6	malaria_status_sobj3Malaria+ at V5	1.0066 (0.6472, 1.5657)	0.9765
		TRT03P_fmRNA 1273.222	0.9814 (0.6576, 1.4647)	0.9264
	Univariate 7	malaria_status_sobj3Malaria+ at V5	1.0302 (0.6594, 1.6095)	0.8956
		SCOV2M6_fVaccine	0.8265 (0.4784, 1.4277)	0.4926

Note:
Covariates besides malaria status included in univariate models have prevalence >=5% in all categories for the cohort.
